#  Anti-tumor activity of Shikonin against afatinib resistant non-small cell lung cancer via negative regulation of PI3K/Akt signaling pathway

**DOI:** 10.1042/BSR20181693

**Published:** 2018-12-11

**Authors:** Baiying Li, Zuguo Yuan, Jin Jiang, Yuanquan Rao

**Affiliations:** 1 Department of Tuberculosis, Hangzhou Red Cross Hospital, Hangzhou, Zhejiang 310003, P.R. China; 2 Department of Radiotherapy, Yinzhou People’s Hospital, Ningbo, Zhejiang 315040, P.R. China; 3 Department of Medical Oncology, Jiaxing Key Subject of Medicine 04-F-14, The First Hospital of Jiaxing, Jiaxing, Zhejiang 314000, P.R. China; 4 Department of Oncology, Zhejiang Hospital, Hangzhou, Zhejiang 310013, P.R. China

**Keywords:** Afatinib-resistance, Apoptosis, Akt, NSCLC, PI3K, Shikonin

## Abstract

Acquired resistance of afatinib is a significant challenge for non-small cell lung cancer (NSCLC) therapy and the mechanisms remain unclear. Aberrant activation of epidermal growth factor receptor (EGFR)-dependent downstream pathways, especially phosphatidylinositol-3-kinases/protein kinase B (PI3K/Akt) signaling pathway has been reported to be involved in the occurrence of afatinib resistance. Developing effective anti-cancer agents to overcome afatinib resistance by targetting PI3K/Akt signaling pathway will be a potential strategy for NSCLC treatment. Shikonin is a naphthoquinone compound isolated from the roots of *Lithospermum erythrorhizon*. In the present study, the anti-cancer activity of Shikonin was evaluated on afatinib-resistant NSCLC *in vitro* and *in vivo*. The data showed that Shikonin inhibited the proliferation and induced apoptosis of afatinib-resistant NSCLC cell line by activating apoptosis signaling pathway and negatively regulating PI3K/Akt signaling pathway. These results revealed that Shikonin was a potential apoptosis inducer in afatinib-resistant NSCLC and a promising candidate for treating patients clinically.

## Introduction

Lung cancer is the most common cause of cancer-related deaths in the world and nearly 80% of lung cancer cases are non-small cell lung cancer (NSCLC). NSCLC is pathologically divided into adenocarcinoma, squamous cell carcinoma, and large cell carcinoma, while adenocarcinoma is the most common pathology type of NSCLC [1]. Epidermal growth factor receptor (EGFR) mutations are involved in the progression of NSCLC and the mutations of EGFR are most frequently found in lung adenocarcinoma cells. The first-generation EGFR tyrosine kinase inhibitor (EGFR-TKI) reversibly binds to the ATP-binding pocket of EGFR, resulting in EGFR signaling inhibition and tumor growth suppression [2]. Erlotinib and gefitinib were the initial TKIs to be approved for NSCLC and showed improved response rates compared with chemotherapy [3].

Afatinib is the second-generation EGFR-TKI that belongs to the irreversible ErbB family blockers [4]. In clinical studies, afatinib provided prolonged survival when compared with chemotherapy, as well as gefitinib as first-line therapy in lung adenocarcinoma and erlotinib in the second-line setting in squamous cell carcinoma (SCC) [5–7]. However, the acquired resistance of afatinib is a significant obstacle for NSCLC therapy and the mechanisms remain unclear. Previous studies have reported that activation of EGFR-dependent downstream pathways, such as phosphatidylinositol-3-kinases/protein kinase B (PI3K/Akt) and mitogen-activated protein kinase/extracellular regulated protein kinases (MAPK/ERK) signaling pathways, might be involved in the occurrence of afatinib resistance [8]. In addition, fibroblast growth factor receptors 1 (FGFR1) activation, amplified KRAS or NRAS expression, also contributed to afatinib resistance [9–11]. Therefore, developing effective anti-cancer agents to overcome afatinib resistance by targetting EGFR-dependent downstream pathways will be a potential strategy for NSCLC treatment.

It has been demonstrated that the agents derived from natural products exhibit potential anti-cancer activity by targetting various signaling pathways [12–21]. Shikonin, a naphthoquinone compound, is isolated from the roots of *Lithospermum erythrorhizon* which has the activities in anti-inflammatory, antioxidation, anti-cancer, and antibiosis. Recently, Shikonin has been studied with its therapeutic effects in malignancies, such as lung caner, lymphoma, breast cancer, esophageal cancer, and so on [22–25]. Shikonin could induce apoptosis, enhance chemotherapeutic sensitivity through targetting of protein tyrosine kinases, and DNA topoisomerases [26,27]. However, the anti-cancer effects and mechanisms of shikonin against afatinib resistant NSCLC remain elusive.

In the present study, we evaluated the anti-cancer activity of Shikonin on afatinib-resistant NSCLC *in vitro* and *in vivo*. The data showed that Shikonin inhibited the proliferation and induced apoptosis of afatinib-resistant NSCLC cell line by activating apoptosis signaling pathway and negatively regulating PI3K/Akt signaling pathway. These results revealed that Shikonin was a potential apoptosis inducer in afatinib-resistant NSCLC and a promising candidate for treating patients clinically.

## Materials and methods

### Chemicals and reagents

Shikonin [5,8-dihydroxy-2-(1-hydroxy-4-methylpent-3-enyl)naphthalene-1,4-dione] was purchased from Sigma–Aldrich (St. Louis, MO, U.S.A.) and dissolved in DMSO (Sigma–ldrich, St. Louis, MO, U.S.A.). Afatinib was obtained from Selleckchem (Houston, TX, U.S.A.). Annexin V Apoptosis Detection Kit was purchased from BD Biosciences (Franklin Lakes, NJ, U.S.A.). MTT was purchased from Sigma–Aldrich (St. Louis, MO, U.S.A.). Antibodies against PI3K, p-PI3K, Akt, p-Akt, Bax, Bcl-2, and cleaved caspase-3 were purchased from Cell Signaling Technology (Danvers, MA, U.S.A.).

### Cell line

The afatinib-resistant NSCLC cell line (H1650/R, H1975/R) was established by the exposure of H1650 and H1975 cells (Cellbank of Chinese Academy of Sciences, Shanghai, China) to afatinib according to the method as previously described [8]. The H1650 and H1975 cells were initially exposed to increased concentrations of afatinib (0.5–5 μM) for 6 months. The resistant clones were selected and maintained under higher concentration of afatinib (1 μM). The resistance persisted when the afatinib was withdrawn. The cells were cultured in RPMI 1640 medium (Gibco, Waltham, MA, U.S.A.) supplemented with 10% FBS (GE Healthcare Life Sciences, Logan, UT, U.S.A.) in a humidified incubator at 37°C containing 5% CO_2_.

### MTT assay

The cells were seeded in 96-well plates at a concentration of 1 × 10^4^/well for 24 h and then exposed to the drugs for 24, 48, and 72 h. MTT solution (5 mg/ml) was added and further incubated for 4 h. Then the solution absorbance was detected at 550 nm wavelength under multi scanner auto reader (Bio-Rad Laboratories, Inc., Hercules, CA, U.S.A.).

### Apoptosis assay

The cells were seeded in six-well plates at a concentration of 5 × 10^4^/well and incubated overnight, then exposed to the drugs for 48 h. After treatment, cells were harvested and washed twice with binding buffer. The apoptosis assay was performed according to the manufacturer’s instructions. Briefly, samples were stained with 5 μl Annexin V-FITC and 5 μl PI and gently mixed prior to incubation for 15 min at room temperature. Apoptosis quantitation was analyzed by FACSCalibur flow cytometer (Becton-Dickinson, San Jose, CA, U.S.A.).

### Western blot analysis

After treatment, the cells were collected, prepared, and washed with ice-cold PBS, then lysed with lysis buffer. Lysates were centrifuged and protein concentrations were determined using BCA kit. Equal amounts of protein were resolved and separated by electrophoresis on SDS/PAGE gel and transferred on to PVDF membranes (Thermo Fisher Scientific Inc., Waltham, MA, U.S.A.). The membranes were then blocked with 5% non-fat milk in TBST (Sigma–Aldrich Co., St. Louis, MO, U.S.A.) prior to further incubation with primary antibodies against PI3K, p-PI3K, Akt, p-Akt, Bax, Bcl-2, and cleaved caspase-3 (1:1000) at 4°C overnight. Then the membranes were washed with TBST and incubated with the HRP–conjugated secondary antibody (1:10000) for 2 h at room temperature. Bands were ultimately visualized with an ECL system (Bio-Rad Laboratories, Inc., Hercules, CA, U.S.A.).

### Animal study

The animal study was carried out according to the NIH guidelines for animal care and use. The study protocol was approved by Ethics Committee of Zhejiang Hospital (Hangzhou, China). The H1650/R cells were washed and resuspended in RPMI 1640 at a concentration of 5 × 10^7^ cells/100 μl. Then the cells were subcutaneously injected into the left flank of nude mice (4-week-old female BALB/c nude mice, Shanghai SLAC Laboratory Animal Co.,Ltd, Shanghai, China) to establish afatinib-resistant NSCLC xenograft. Tumor volumes were calculated by the formula: 0.5 × length × width^2^. The xenograft tumors were grown for 12 days to reach an average volume of 0.2 cm^3^ and then the mice were intraperitoneally injected with Shikonin (2 mg/kg) daily, and the mice in the control group were intraperitoneally injected with 0.1 ml PBS daily. Tumor volumes were measured weekly and the mice were killed at the end of 4 weeks. Tumors were taken for further evaluation.

### Immunohistochemistry

Tumor tissues were embedded in paraffin and sectioned. The sections (4 μm) were deparaffinized in xylene, hydrated with different concentrations of ethanol, and then blocked by endogenous peroxidase. The sections were pretreated in citrate buffer for antigen retrieval. Immunostaining was undertaken by incubation with primary antibody (anti-p-Akt, 1:100; anti-Bax, 1:100; anti-Bcl-2, 1:400; anti-cleaved caspase-3, 1:1000) at 4°C overnight and then HRP-labeled second antibody at room temperature for 1 h. The sections were incubated with DAB substrate according to manufacturer’s instructions. Immunostaining results were evaluated with light microscopy under 200× magnification by two pathologists.

### Statistical analysis

Data were presented as mean ± S.D. One-way ANOVA and SNK-q test were performed to determine differences between groups using SPSS 19.0 software (SPSS Inc., Chicago, IL, U.S.A.). *P*<0.05 was considered to indicate a significant difference.

## Results

### Shikonin inhibited the proliferation of afatinib-resistant NSCLC cell lines

**Figure 1:**
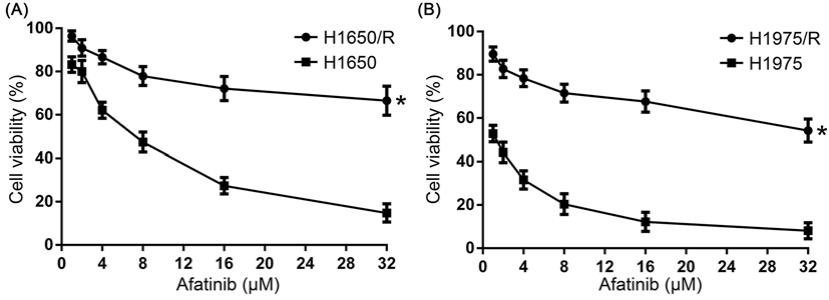
The proliferation of afatinib-resistant NSCLC cell lines under afatinib treatment (**A**) H1650/R cells showed significant resistance to afatinib compared with H1650 cells. (**B**) H1975/R cells showed significant resistance to afatinib compared with H1975 cells. *Statistically significant difference (*P*<0.05).

**Figure 2:**
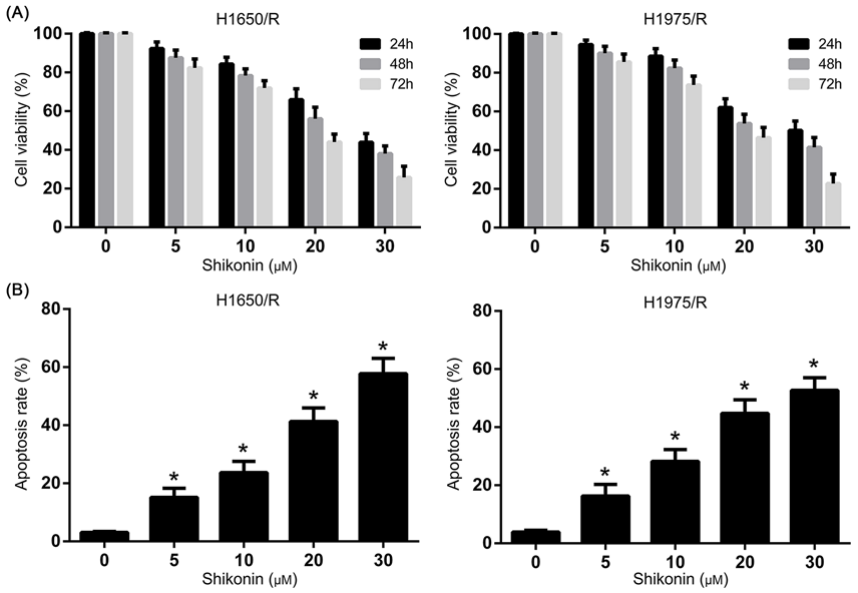
The anti-tumor activity of Shikonin on the proliferation of afatinib-resistant NSCLC cell lines (**A**) Shikonin inhibited the proliferation of H1650/R and H1975/R cells in a dose- and time-dependent manner. (**B**) Shikonin significantly induced apoptosis of H1650/R and H1975/R cells in a dose-dependent manner compared with control group. *Statistically significant difference compared with control group (*P*<0.05).

The anti-tumor activity of Shikonin on the proliferation of afatinib-resistant NSCLC cell lines H1650/R and H1975/R was determined by MTT assay. H1650/R and H1975/R cells showed significant resistance to afatinib compared with their parental cells that were treated with increasing concentrations (1–32 μM) of afatinib for 48 h (Figure 1A,B). Further, H1650/R and H1975/R cells were treated with increasing concentrations (1–20 μM) of Shikonin for 24, 48, and 72 h and the result showed that Shikonin significantly inhibited the proliferation of H1650/R and H1975/R cells in a dose- and time-dependent manner (Figure 2A).

### Shikonin induced apoptosis of afatinib-resistant NSCLC cell lines

To investigate the effect of Shikonin on apoptosis, Annexin V-FITC apoptosis assay was performed to detect the apoptosis rate in H1650/R and H1975/R cells. The H1650/R and H1975/R cells were exposed to Shikonin for 48 h and the data from flow cytometry analysis showed that Shikonin significantly induced apoptosis in a dose-dependent manner compared with control group (Figure 2B). The result revealed that Shikonin-mediated suppression of cell viability in afatinib-resistant NSCLC cells was correlated with enhanced apoptosis induction.

### Shikonin activated apoptosis signaling pathway and negatively regulated PI3K/Akt signaling pathway of afatinib-resistant NSCLC cell lines

**Figure 3:**
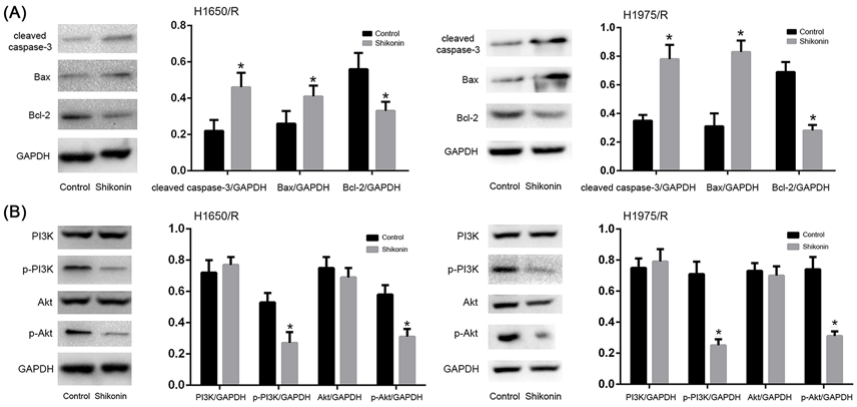
Shikonin regulated PI3K/Akt signaling pathway of afatinib-resistant NSCLC cell lines (**A**) Shikonin increased the expression of cleaved caspase-3 and Bax, while decreased Bcl-2 expression of H1650/R and H1975/R cells compared with control group. (**B**) Shikonin down-regulated the expressions of p-PI3K, p-Akt of H1650/R and H1975/R cells compared with control group. *Statistically significant difference compared with control group (*P*<0.05).

Apoptosis is a type of programmed cell death which is caspase-dependent and correlated with Bax/Bcl-2 balance. The H1650/R and H1975/R cells were exposed to Shikonin for 48 h to investigate the expression of apoptosis related key proteins. The levels of cleaved caspase-3 and pro- and anti-apoptotic mitochondrial proteins Bax, Bcl-2 were detected by Western blot. The result showed that Shikonin increased the expression of cleaved caspase-3 and Bax, while decreased Bcl-2 expression compared with control group in H1650/R and H1975/R cells (Figure 3A), suggesting that Shikonin could induce apoptosis by activating apoptosis signaling pathway.

PI3K/Akt signaling pathway plays important role in afatinib resistance, as it regulates cell apoptosis, tumor invasion, metastasis, and drug resistance. Thus, targetting PI3K/Akt signaling is very critical for lung cancer therapy. The H1650/R and H1975/R cells were exposed to Shikonin for 48 h and the result showed that the expressions of p-PI3K, p-Akt were down-regulated compared with control group in H1650/R and H1975/R cells (Figure 3B), indicating that Shikonin inhibited cell viability and induced apoptosis of afatinib-resistant NSCLC cells via negative regulation of PI3K/Akt signaling pathway.

### Shikonin suppressed tumor growth *in vivo*

**Figure 4:**
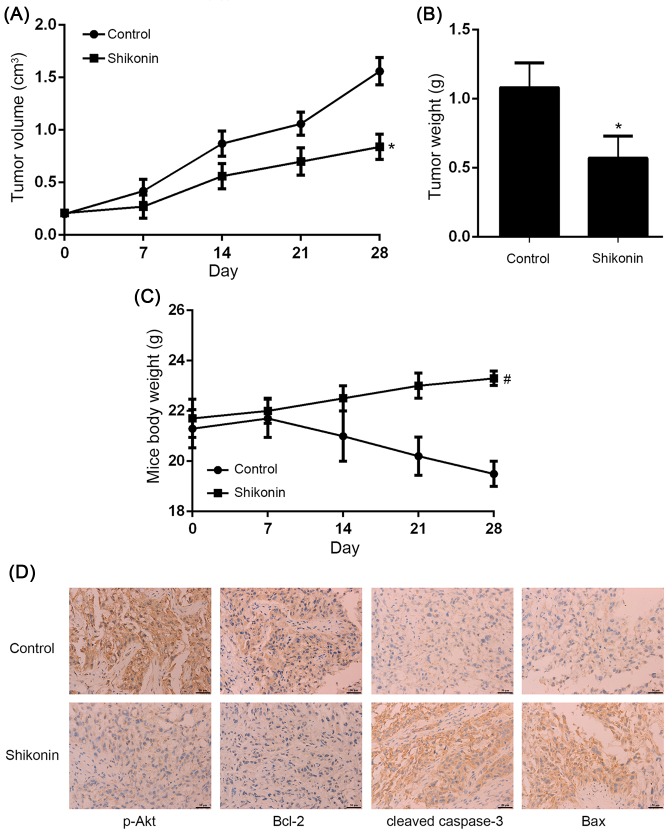
Shikonin suppressed afatinib-resistant NSCLC xenograft tumor growth *in vivo* (**A**,**B**) The volume and weight of H1650/R xenograft tumors were significantly suppressed by Shikonin compared with control group. (**C**) The mice body weight exhibited no significant change between Shikonin and control group. (**D**) Immunohistochemical analysis showed Shikonin decreased p-Akt and Bcl-2 expression, while increased cleaved caspase-3 and Bax expression *in vivo*. *Statistically significant difference compared with control group (*P*<0.05). ^#^No statistically significant difference compared with control group (*P*>0.05).

The volume and weight of H1650/R xenograft tumors were significantly suppressed by Shikonin compared with control group (Figure 4A,B). The cytotoxic effect of Shikonin was also evaluated and it showed that mice body weight exhibited no significant change between Shikonin and control group (Figure 4C). To preliminarily explore the underlying mechanism of Shikonin against afatinib-resistant NSCLC *in vivo*, immunohistochemical analysis was performed and the result indicated that Shikonin decreased p-Akt and Bcl-2 expression, while increased cleaved caspase-3 and Bax expression (Figure 4D), confirming that Shikonin suppressed afatinib-resistant NSCLC xenograft tumor growth by regulating PI3K/Akt signaling pathway *in vivo*.

## Discussion

Shikonin is a naphthoquinone isolated from natural herb and has been used for long history in east Asia due to its efficacy and safety. Previous studies have confirmed its anti-cancer activities in numerous malignancies and explored the underlying mechanisms. Shikonin inhibited gallbladder cancer cells by inducing apoptosis and G_0_/G_1_ phase arrest via the c-Jun N-terminal kinase (JNK) signaling pathway [28]. Shikonin induced glioma cell necroptosis by ROS overproduction and promoting receptor-interacting protein 1 (RIP1)/RIP3 necrosome formation [29]. Shikonin induced apoptosis through ROS/JNK signaling in cholangiocarcinoma cells [30]. Shikonin inhibited migration and invasion of lung caner via suppression of c-Met mediated epithelial–mesenchymal transition (EMT) [31]. Shikonin induced apoptosis of lung cancer cells via activation of Forkhead box O3 (FOXO3a)/early growth response protein 1 (EGR1)/Sirtuin 1 (SIRT1) signaling antagonized by p300 [32]. Shikonin derivative DMAKO-05 inhibited Akt activation and melanoma proliferation [33]. Shikonin induced cervical cancer cell apoptosis via PI3K/Akt signaling [34]. Shikonin also showed powerful cytotoxic effects in drug resistant cancers. It has been reported that Shikonin inhibited gefitinib-resistant NSCLC by inhibiting Thioredoxin reductases (TrxR) and activating the EGFR proteasomal degradation pathway [35]. Shikonin exhibited anti-cancer effects on multidrug-resistant hepatocellular carcinoma [36]. However, studies about the anti-cancer effects of Shikonin and associated mechanisms in afatinib-resistant NSCLC are still unclear and insufficient.

Here, the activity of Shikonin against afatinib-resistant NSCLC was evaluated *in vitro* and *in vivo*. In the present study, we first established afatinib-resistant NSCLC cell line (H1650/R, H1975/R), and the MTT assay showed that Shikonin significantly inhibited the proliferation of afatinib-resistant H1650/R and H1975/R cells in a dose- and time-dependent manner. Meanwhile, Shikonin significantly induced apoptosis in a dose-dependent manner compared with control group in H1650/R and H1975/R cells, suggesting Shikonin-mediated suppression of cell viability in afatinib-resistant NSCLC cell was correlated with enhanced apoptosis inducing. In *in vivo* study, the growth of H1650/R xenograft tumors were also significantly suppressed by Shikonin compared with control group and mice body weight exhibited no significant change.

The PI3K/Akt signaling pathway is one of the most important pathways in cancer development, and it has been demonstrated that aberrant activation of the PI3K/Akt signaling promoted TKI-resistance in NSCLC [37–39]. Therefore, inhibition of the PI3K/Akt pathway by effective anti-cancer agents might overcome TKI resistance. Recent studies reported that Polyphyllin II restored the sensitization of gefitinib-resistant NSCLC by negatively regulating PI3K/Akt signaling pathway [40]. miR-223 reversed the resistance of erlotinib through insulin-like growth factor 1 receptor (IGF1R)/PI3K/Akt signaling pathway [41]. BMS-708163 reversed resistance to gefitinib via the PI3K/Akt pathway in lung cancer [42]. However, the mechanism of acquired resistance of afatinib remains unclear. Previous studies indicated that afatinib resistance resulted in markedly decreased expression of EGFR family proteins including EGFR, HER2, and HER3, but exhibited higher activation of Akt [43,44]. The present study demonstrated that the tumor suppression by Shikonin was associated with activated apoptosis signaling pathway and negatively regulated PI3K/Akt signaling pathway. Shikonin increased the expression of cleaved caspase-3 and Bax, while decreased Bcl-2 expression compared with control group in afatinib-resistant NSCLC cells, suggesting that Shikonin could induce apoptosis by activating apoptotic signaling pathway. Further, the expressions of p-PI3K and p-Akt were down-regulated significantly by Shikonin treatment in afatinib-resistant NSCLC cells, demonstrating that Shikonin inhibited cell viability and induced apoptosis of afatinib-resistant NSCLC cells via negative regulation of PI3K/Akt signaling pathway. The results of *in vivo* study further confirmed that Shikonin suppressed afatinib-resistant NSCLC xenograft tumor growth by decreasing p-Akt, Bcl-2 expression and increasing cleaved caspase-3, Bax expression.

In summary, our results demonstrated that Shikonin showed definite anti-cancer activity of afatinib-resistant NSCLC. The anti-proliferative effect of Shikonin was associated with apoptosis inducing by negative regulation of PI3K/Akt signaling pathway. Therefore, Shikonin may be an effective anti-cancer candidate in the clinical settings of afatinib-resistant NSCLC. The preliminary molecular mechanism of Shikonin on the afatinib-resistant NSCLC was revealed and it is an important foundation for further studies.

## Abbreviations

EGFR: epidermal growth factor receptor
EGFR-TKI: EGFR tyrosine kinase inhibitor
JNK: c-Jun N-terminal kinase
NSCLC: non-small cell lung cancer
PI3K/Akt: phosphatidylinositol-3-kinases/protein kinase B
